# Multimode Gesture Recognition Algorithm Based on Convolutional Long Short-Term Memory Network

**DOI:** 10.1155/2022/4068414

**Published:** 2022-03-02

**Authors:** Ming-Xing Lu, Guo-Zhen Du, Zhan-Fang Li

**Affiliations:** ^1^Department of Public Studies, Henan Vocational College of Nursing, Anyang 455000, China; ^2^School of Continuing Education, China University of Mining and Technology, Xuzhou 221008, China

## Abstract

Gesture recognition utilizes deep learning network model to automatically extract deep features of data; however, traditional machine learning algorithms rely on manual feature extraction and poor model generalization ability. In this paper, a multimodal gesture recognition algorithm based on convolutional long-term memory network is proposed. First, a convolutional neural network (CNN) is employed to automatically extract the deeply hidden features of multimodal gesture data. Then, a time series model is constructed using a long short-term memory (LSTM) network to learn the long-term dependence of multimodal gesture features on the time series. On this basis, the classification of multimodal gestures is realized by the SoftMax classifier. Finally, the method is experimented and evaluated on two dynamic gesture datasets, VIVA and NVGesture. Experimental results indicate that the accuracy rates of the proposed method on the VIVA and NVGesture datasets are 92.55% and 87.38%, respectively, and its recognition accuracy and convergence performance are better than those of other comparison algorithms.

## 1. Introduction

Human-computer interaction systems are a bridge between humans and machines for communication and information transmission [1]. Traditional human-computer interaction requires the use of mouse, touch screen, camera, and other input devices. The form of information conveyed has also evolved from the early encoded characters to the form of images and videos. With the development of science and technology and people's increasing application needs for smart devices, recognizing limb movements through machines has become one of the hot research directions [2].

Gesture recognition methods are mainly divided into two methods: data-based gloves and video-based data. Literature [3] invented the data glove, which contains sensor devices that convert physical information such as hand movement posture into digital information for use by computers and perform gesture recognition. However, data gloves are expensive and overreliant on auxiliary devices, and the user experience is not good, which is difficult to promote. In a visual data-based approach, users do not need to wear devices such as data gloves. They are only equipped with a camera to achieve gesture recognition, and the accuracy and speed of recognition are within the acceptable range.

Traditional common models of vision-based dynamic gesture recognition include the hidden Markov model (HMM) [4] and the Dynamic Time Regularization (DTW) model [5]. Literature [6] proposes a gesture recognition model that integrates global gesture movement and finger local movement and extracts keyframes according to joint coordinates and distance functions, using support vector machines to achieve dynamic gesture recognition and classification. Literature [7] proposes establishing a recognition model for gestural three-dimensional trajectory feature vectors and hand-shaped features and then fuses the recognition results. Although the model can improve the accuracy, the trajectory feature acquisition steps are numerous and the processing is complex. Overall, traditional models often require manual extraction of features. The ability to characterize a small number of features is relatively insufficient, and manual extraction of complex features is very difficult, resulting in a situation based on the poor performance of traditional gesture recognition models and evaluation indicators.

In recent years, deep learning has been widely used in areas such as object recognition and classification tasks. Traditional 2D convolutional networks have strong feature extraction capabilities for images, but they do not capture the timing relationship between images very well. Therefore, it is extremely easy to lose target information in the processing of continuous video frames. Literature [8] proposed a 3D convolutional network (3DCNN) for human behavior recognition, which overcomes the shortcomings of traditional 2DCNN in video processing. In literature [9], based on the 3D convolutional neural network, a three-dimensional CNN model is proposed and the performance is obtained, which mainly proposes a structure for extracting spatiotemporal features from video clips. In literature [10], multidirectional 3D convolutional neural networks are used for feature fusion and gesture recognition. In order to avoid the impact of redundant data in gesture video on network accuracy, it uses optical flow method to key frame extraction of video. Although the experimental results obtained by this model are better than those of the uniform sampling method, the model needs to calculate the optical flow per frame. It is expensive to calculate in actual use or on long videos, and its range of adjustable parameters is limited.

At present, the existing isolated gesture recognition model based on deep learning has a high recognition rate. Most of the methods are based on recurrent neural networks (RNNs) [11]. Then, more and more novel and efficient network architectures are being proposed. One of the more representative methods is the dense convolutional neural network proposed in literature [12], which has a deeper network hierarchy than traditional CNNs. The convolutional layers within its modules are densely related to each other, so that the network has a deep hierarchy, while avoiding the problem of information loss due to the network being too deep. Experimental results show that DenseNet has high feature extraction ability and recognition rate. For complex gestures, 3D CNNs can effectively learn the space, structure, and posture transformation of gestures in successive video frames within the video, which is what two-dimensional CNNs of a single-frame image or picture lack. In the traditional 3D CNNs model training process, with video clips (shorter consecutive frames) as input information, there will often be repeated inputs. If the repeat section is large, the training speed of the model will be greatly longer. Therefore, how to simplify learning operations and efficiently train models is a very important topic.

For time series models, literature [13] proposes a new structure for solving sequence problems, namely, time convolutional neural networks (TCNs). TCNs have better clarity and simplicity than traditional RNNs and their typical recursive architecture LSTMs and GRUs. In order to extract more complete and representative feature information, literature [14] proves that there are multiple relationships within feature information in neural networks, proposing to use the attention mechanism as an embedded module for deep learning models. The compression-stimulus network SENets [15] is an efficient architectural unit based on attention mechanisms. The goal is to improve the quality representation generated by the network by explicitly modeling the interdependencies between their convolutional feature channels.

The innovations and contributions of this paper are listed in the following:

1. Aiming to further improve the recognition performance of multimodal gestures, this paper proposes a multimodal gesture recognition method based on convolutional long-term memory network.
2. The data features are automatically extracted by convolutional neural networks. Long short-term memory (LSTM) is applied to learn the relevance advantages of time series data, and the SoftMax classifier is used to realize gesture recognition.

The structure of this paper is listed as follows. Related studies are described in the next section. The proposed gesture recognition mode is expressed in Section 3.  Section 4 focuses on the experiment and analysis.  Section 5 is the conclusion.

## 2. Related Studies

Vision-based gesture recognition techniques include methods for static gestures and methods for dynamic gestures [16]. In recent years, CNNs have made major breakthroughs in computer vision-related tasks with their powerful feature extraction capabilities, so the features extracted by CNNs have been widely used in many action classification tasks to obtain better performance. Two-dimensional convolutional neural networks (2D-CNNs) were originally applied to two-dimensional images, that is, single-frame images in static gestures or dynamic gesture videos, such as literature [17] using two-dimensional CNNs and identifying image gestures through multilayer hierarchical pooling to extract information in space and time domain. The development of three-dimensional convolutional neural networks (3D-CNNs) has made three-dimensional convolution (C3D) widely used in subsequent studies. Literature [18] introduces three-dimensional CNNs into dynamic video gesture recognition with great performance. The main contribution of the study was to propose an architecture for extracting spatiotemporal features from video footage. On the other hand, literature [19] designed a multistream 3D-CNNs classifier for gesture recognition. The classifier consists of two subnets, a high-resolution network (HRN) and a low-resolution network (LRN). Literature [20] proposes a new time pooling method in order to solve the problem of gesture fragment training in video.

With the development of deep convolutional neural networks, more and more CNNs architectures have been proposed, such as AlexNets [21], VGGNets [22], GoogleNets [23], ResNets [24], and SenseNets [25]. The goal of the above model is to build a higher-level CNNs architecture. These models mine deeper, more complete statistical features from low-level image frames and then classify them. In the field of isolated gesture recognition, literature [26] uses the Res-C3D model for application in gesture recognition tasks. Literature [27] also applies to the Res-C3D model and twice won first place in the ChaLearn LAP Multimodal Isolated Gesture Recognition Challenge in 2016 and 2017. This is enough to prove that the deeper the network, the greater ability to learn features. As one of the latest convolutional structures, SenseNets is gradually being applied to motion recognition, especially face recognition and gesture recognition. In addition to the field of image recognition, in recent studies, DenseNets has also been used to classify different behaviors, such as literature [28] using SenseNets for behavior recognition studies. Depth information is used by domestic and foreign research institutes as additional video information in addition to RGB information, and its Chinese [29] use depth maps to identify gestures.

For temporal information of video sequences, LSTM networks are a common choice for gesture recognition. For example, literature [30] introduces convolutional long short-term memory (conv-LSTM) models into spatiotemporal feature maps to identify them by the relationships between before and after in gesture videos. The Literature [31]uses 2S-RNN (based on RGB and depth maps) for continuous gesture recognition. However, RNNs, including LSTMs and GRUs, have shortcomings in the time domain such as short-term information learning and excessive storage capacity. To compensate for these deficiencies, TCNs were proposed and applied to gesture reproduction. Literature [32] proposes res-TCNs, a skeleton-based dynamic gesture recognition method. The experimental results show that, compared with traditional RNNs, TCNs are more concise in structure and can effectively improve the recognition rate. There is a lot of redundant information in the whole time series data, so it is important to introduce attention mechanisms. While using the time series model in literature [33], the relevant attention mechanism model is embedded, which reduces the error rate on the basis of the recognition rate of the original time series model.

## 3. Gesture Recognition Model

### 3.1. CNN Model

Although deep learning networks have achieved good results in the fields of image classification, face recognition, and natural language processing, they have not yet obtained a recognized structure in the application of sequence signal classification. Therefore, this paper designs a CNN model for gesture recognition based on LeNet-5. Compared with LeNet-5, the input layer data format of the CNN model in this paper is 24 × 410 sequences, and batch normalization layer and Leaky ReLU are added after each maximum pooling layer. The CNN structure mainly includes the sequence input layer, the folding layer, the CNN feature extraction layer (Convolution Maxpooling Batch Normalization Leaky ReLU), the defolding layer, the flatten layer, the fully connected layer, and the SoftMax classification layer. The CNN model contains a total of three CNN feature extraction layers. The convolutional layer of each feature extraction layer extracts gesture data features and is a key layer of the CNN model. The maximum pooling layer compresses data and reduces dimensions. The batch normalization layer normalizes the extracted features. The nonlinear activation layer (Leaky ReLU) facilitates the mapping of features after batch normalization. A fully connected layer reduces the loss of information for extracted features. The SoftMax classification layer ultimately implements the classification of gestures.

Google proposed batch normalization technology in 2015, which is applied to deep neural network training not only to accelerate the convergence speed of the model but also to a certain extent to alleviate the problem of “gradient diffusion” in the deep network, making the trained deep learning model more stable. The Leaky ReLU activation function is to solve the problem that occurs when the ReLU input value is negative. The output is always 0, and the first derivative is always 0. It results in neuronal parameters not being updated and neurons not learning, as defined in the following equation:

(1) $$\begin{array}{c} f\left(i\right)=\left\{\begin{array}{c} i,\;i\geq 0,\\ s\times i,\;i<0, \end{array}\right. \end{array}$$

where *s* is a nonnegative number not less than 1, and when *s* takes 0, the Leaky ReLU activation function degenerates into a ReLU function. The SoftMax classification layer is shown in (2).

(2) $$\begin{array}{c} s\left(i_{x}\right)=\frac{e^{i_{x}}}{\sum_{y=1}^{z}e^{i_{y}}},\;x=1,2,\ldots ,z, \end{array}$$

where *i*_*x*_ is the feature sequence of the extracted gesture data. *Z* is the number of gesture categories. The classification result of the SoftMax function represents the probability of ownership of the input sample when it is divided into each category, and the sum of the probabilities of belonging is 1.

Since CNN can automatically extract the deep features of gesture data, which can avoid many problems caused by manual feature extraction, this paper introduces the CNN feature extraction layer as the feature extraction unit of the proposed CLT-Net network model. The specific process of CNN feature extraction layer to achieve feature extraction is that the numbers of convolutional nuclei of the three convolutional layers are set to 32, 128, and 32 in turn, and the convolutional kernel size is set to (1, 11), (1, 9), and (1, 7), and the step size is (1, 2); that is, the vertical step is 1, the horizontal step is 2, and the model adopts the same method for “padding.” The pooling kernel size of the three maximum pooling layers is (1, 3) and the step size is (1, 2). When the input data scale of a single sample is 24 × 410 × 1, the scales of the gesture feature sequence obtained by the three CNN feature extraction layers are 24 × 102@32, 24 × 25@128, and 24 × 6@32, respectively.

### 3.2. LSTM Model

**Figure 1:**
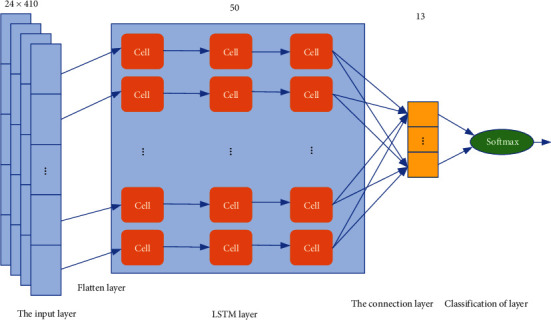
The structure of LSTM.

LSTM is an improvement on the recurrent neural network (RNN), which was proposed in 1997 [34]. The core part of the LSTM network is the sequence input layer and the LSTM layer. The sequence input layer can input sequence or time series data into the network, and the LSTM layer can learn the long-term dependencies between the time steps of the sequence data, which is a good solution to the problem of disappearing RNN gradients [35]. Since LSTM is a powerful time series signal processing and prediction method and gesture sensor data belongs to the signal on the time series, this paper introduces the LSTM layer as a feature filtering unit of the proposed CLT-Net network model. The LSTM model structure is shown in Figure 1, which mainly includes the sequence input layer, flatten layer, LSTM layer, full connection layer, and SoftMax classification layer.

As can be seen from Figure 1, the sample size of the sequence input layer is 24 × 410 × 1, and the multidimensional data is one-dimensionalized as the LSTM layer input after the flatten layer, the number of hidden elements of the LSTM layer is set to 50, the hidden node of the fully connected layer is set to 13, and finally the SoftMax classification layer implements the classification of different gestures.

**Figure 2:**
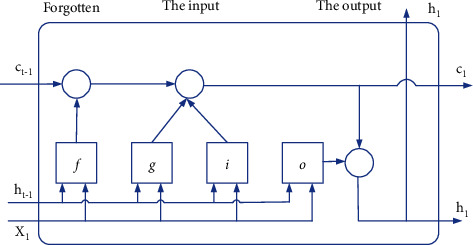
LSTM cell internal structure.

Cells in the LSTM layer provide time dependence on the input data, giving the data time characteristics, and the LSTM network achieves long-term control through the cells, which in turn is used for classified prediction of timing signals. Cell function is mainly achieved through the forgetting gate, input gate, and output gate. The internal structure of the LSTM layer cells is shown in Figure 2.

The LSTM layer learns weights as input weight *M*, recursive weight *R*, and bias *h*. Matrices *M*, *R*, and *h* are a series of input weights, recursive weights, and deviations for each component, respectively, as shown as follows:

(3) $$\begin{array}{c} M=\left[\begin{array}{c} M_{x}\\ M_{f}\\ M_{a}\\ M_{o} \end{array}\right],\\ \;R=\left[\begin{array}{c} R_{x}\\ R_{f}\\ R_{a}\\ R_{o} \end{array}\right],\\ \;h=\left[\begin{array}{c} h_{x}\\ h_{f}\\ h_{a}\\ h_{o} \end{array}\right], \end{array}$$

where *t*-moment cell state output and hidden state output are shown in the two following formulas:

(4) $$\begin{array}{c} c_{n}=f_{n}⊙c_{n-1}+x_{n}⊙a_{n}, \end{array}$$

(5) $$\begin{array}{c} b_{n}=o_{n}⊙\sigma_{c}\left(c_{n}\right), \end{array}$$

where ⊙ is the Hadamard product (multiplication of elements of vectors). *σ*_*c*_ is a hyperbolic tangent function (tanh) state activation function. In Figure 2, *t*-moment forgetting activation *f*_*n*_, input activation *x*_*n*_, output activation *o*_*n*_, and candidate unit input *a*_*n*_ as shown in the four following equations:

(6) $$\begin{array}{c} f_{n}=\sigma_{a}\left(M_{f}i_{n}+R_{f}b_{n-1}+h_{f}\right), \end{array}$$

(7) $$\begin{array}{c} x_{n}=\sigma_{a}\left(M_{x}i_{n}+R_{x}b_{n-1}+h_{x}\right), \end{array}$$

(8) $$\begin{array}{c} o_{n}=\sigma_{a}\left(M_{o}i_{n}+R_{o}b_{n-1}+h_{o}\right), \end{array}$$

(9) $$\begin{array}{c} a_{n}=\sigma_{c}\left(M_{a}i_{n}+R_{a}b_{n-1}+h_{a}\right), \end{array}$$

where *b*_*n*−1_ is the output information of the previous hidden state. *i*_*n*_ is the input information for the current moment. Participate in network training by using *b*_*n*−1_ and *i*_*n*_ as input information for the current time step. After passing through the gate activation function *σ*_*a*_, the output is finally given to a value between [0, 1].

The larger the amnesia activation *f*_*n*_ is, the less *c*_*n*−1_ is output in the cell state at the previous moment of forgetting. The larger the input activation *x*_*n*_, the more information representing the candidate input *a*_*n*_ is written into the current moment; that is, the forgetting activation *f*_*n*_ and the input activation *x*_*n*_ jointly determine the degree of reception of different input information by the output *c*_*n*_ of the cell state at the current moment. The output activation *o*_*n*_ determines the output *b*_*n*_ of the hidden state at the current moment, and the above control strategy implements the long-term dependence of gesture data on the time step sequence.

### 3.3. CLT-Net Gesture Recognition Model

**Figure 3:**
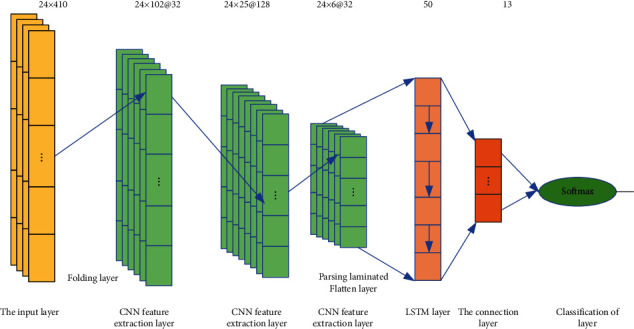
CLT-Net model structure.

Since the gesture data collected by inertial sensors can be regarded as time series signals, the current machine learning algorithms rely heavily on manual design, which may lead to insufficient utilization of information and inability to effectively achieve problems such as complex gesture pose recognition. In this paper, a deep learning model (CLT-Net) based on space-time feature fusion is proposed for gesture recognition. The CLT-Net model fully combines the advantages of the correlation between CNN's automatic extraction of data depth features and LSTM learning time series data, adopts the same network structure as the CNN model, and replaces the first fully connected layer of the CNN model with the LSTM layer. The CLT-Net network model parameters are set and the way the function is selected is consistent with the CNN module and the LSTM module of the corresponding structure, and its structure is shown in Figure 3, which mainly includes the sequence input layer, the folding layer, the CNN feature extraction layer (Convolution Maxpooling Batch Normalization Leakage ReLU), the defolding layer, the flatten layer, the LSTM layer, the fully connected layer, and the SoftMax classification layer.

The classification process of gesture recognition method based on the CLT-Net model is as follows: first input gesture data series, two-dimensional spatial feature extraction through the CNN module, expand the obtained two-dimensional data features into one dimension through the flatten layer, and then enter the LSTM layer to filter the features on the time series, and then the filtered gesture features go through the full connection layer, map to the sample marker space through the weight matrix, and finally classify and calculate through the SoftMax layer. The category with the largest prediction probability is selected as the prediction category for the sample of the input data. The model will be trained according to the error between the prediction category obtained by forward propagation and the real sample label and back propagation according to the loss function and optimizer used by the model to continuously correct the weights and bias terms in the network, and finally the model training is realized and a better model is obtained.

## 4. Experiments and Analysis

All models in this article were trained and tested using a computer configured as a Core i7-8700U CPU @3.20 GHz, with 16 GB of memory. The computer system is 64-bit Windows 11. All models are implemented using the Matlab 2019a Deep Learning Toolbox framework.

### 4.1. Dataset

This section compares the present method with other recent dynamic gesture methods. In this experiment, the model proposed in this paper is evaluated by two publicly available multimodal dynamic gesture datasets.

1. VIVA [32]. The VIVA dataset is a multimodal dynamic gesture dataset specifically designed to study situations of natural human activity in real-world driving environments. It has a total of 885 RGB and depth information video sequences.
2. NVGesture [36]. In order to study the human-machine interface, the NVGesture dataset uses multiple sensors and multiple angles for acquisition, containing 1532 dynamic gestures. In the experiments in this paper, RGB, depth, and optical flow modes will be used as data inputs to the model, respectively. Among them, the optical flow diagram is calculated from the RGB stream using the method proposed in the literature.

### 4.2. Experimental Parameters

The experimental parameters are set as shown in Table 1. The initialization parameters of all models are configured in the simulation experiment so that all models are compared under relatively fair conditions. This is more conducive to accurately reflecting the realism of the CNN model, the LSTM model, and the model in this paper.

The CNN layer and the fully connected layer use the Kaiming method to initialize their weight coefficients, which is conducive to accelerating the convergence speed of the model, while the LSTM layer uses the orthogonal method to initialize the weight coefficients. The optimizer for all models uses the Adaptive Moment Estimation Optimization Algorithm (Adam algorithm) [37], which has faster convergence speeds and lower memory consumption and can be trained without the use of validation sets.

### 4.3. Model Convergence Rate

**Figure 4:**
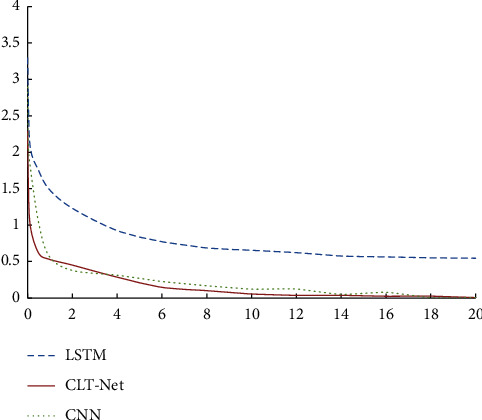
The comparison of convergence speed.

In order to analyze the convergence rate of the model in this paper, the LSTM, CNN, and CLT-Net model loss functions are compared during training, and the curves are shown in Figure 4. As can be seen from Figure 4, as the number of iterations increases during the training process, the loss function curve of the three models gradually approaches 0, indicating that the correction and update of the parameters of the model gradually approach the better values.

Observation of Figure 4 shows that as the number of iterations increases, the loss function of the model tends to stabilize faster, which means that the model in this article has the fastest convergence speed. It makes the classification accuracy and loss function values of the training set close to steady state. With the increase of the number of iterations, the classification accuracy of the model in this paper gradually reaches the highest, and the loss value is gradually reduced to the minimum.

### 4.4. Experiment Results on the VIVA Dataset

Table 2 shows the performance of dynamic gestures tested on two modes of RGB and depth information in the VIVA dataset.

Experimental results show that the proposed method obtains a 92.55% accuracy rate on the VIVA dataset. As can be seen from Table 2, in terms of accuracy, the T3D-Dense + TCNtse proposed in this paper is far superior to HOG + HOG2, CNN : LRN, CNN : LRN : HRN, and C3D methods, which are 27.04%, 17.14%, 14.04%, and 14.14% higher, respectively. The identification accuracy of the proposed method is relatively close to that of I3D and MTUT methods, which is partly due to the fact that the pretraining methods used by I3D and MTUT are similar to those used in this paper. Nonetheless, it can be seen that the performance of the RGB and depth networks in this paper is improved by 8.44% and 5.46% on the basis of I3D and MTUT, respectively.

At the same time, in order to prove the effectiveness of each module, other methods are tested on the VIVA dataset in this paper, and the main methods are as follows:

1. Full 3D-DenseNets. The essence of the 3D-DenseNets pretraining process is that the full 3D-DenseNets recognize dynamic gestures, so the pretrained 3D-DenseNets can be tested directly, and the test recognition accuracy rate is 89.22%.
2. Res3D + TCNs. By changing the backbone framework of the short-term spatiotemporal feature extraction module, T3D-Dense, to Res3D network, it can be found that the basic T3D-Dense as the backbone framework is superior to the Res3D + TCNs network with the Res3D backbone framework. Moreover, the parameter quantity of the T3D-Dense + TCNs network in this paper is only 1.41 million, while the parameter amount of the Res3D + TCNs network is 45.35 million, which is 30 times the parameter amount of T3D-Dense + TCNs, which proves the superiority of the proposed algorithm.
3. The difference between T3D-Dense + TCNs and the proposed method is whether the TSE module is embedded in the TCN network, and it can be seen that the embedded TSE module increases the recognition rate of the network by 0.81%.

**Figure 5:**
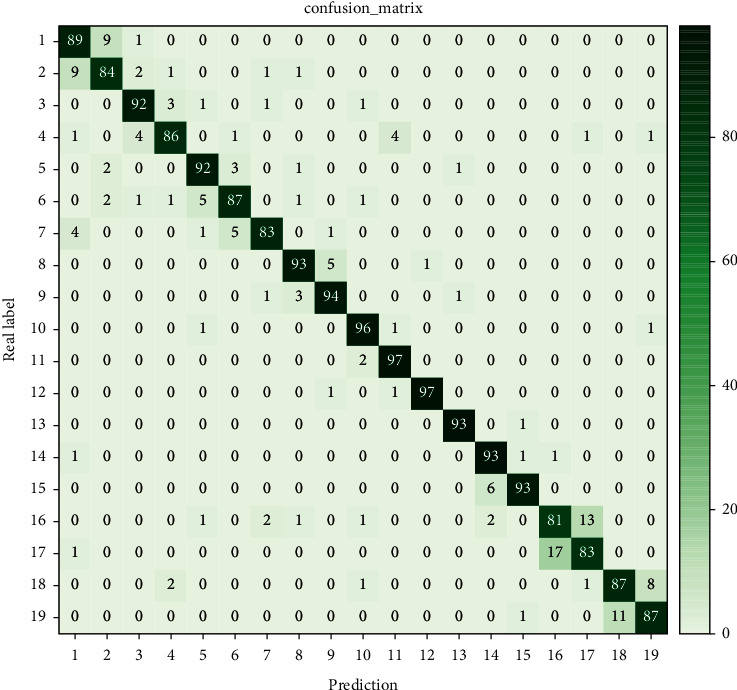
The recognition confusion matrix of the VIVA dataset.

This article counts the confusion matrices of the final classification obtained according to the method in this paper, as shown in Figure 5.

In the experiment, it was found that there was a high misidentification rate between class 1 and class 2 and between class 16 and class 17 on the VIVA dataset; in particular, between class 16 and class 17 (of which class 16 was clockwise circled by gestures, and class 17 was counterclockwise circled), the misidentification rate was 15%. To this end, the weights of the TSE modules in class 16 and class 17 are extracted from each layer of TCN for visualization. Since the structural spatial information of class 16 and class 17 has more similarity in short time, the TSE cannot distinguish between the two well in terms of weight control, so that the two will have a higher misidentification rate in recognition. However, on the weights of most of the gestures, especially on the 5 frames to 12 frames of the third layer of TCNs, there is a large degree of differentiation. Experimental results show that TSE has a good effect on TCNs identification.

### 4.5. Experiment Results on the NVGesture Dataset

In order to test the method of this paper in two or more data flow tasks, RGB + depth information, RGB + optical flow information, and RGB + depth + optical flow information were entered into the NVGesture dataset, respectively, and the classification results are shown in Table 3. The bold font in Table 3 means the best result.

In the RGB + depth information, the proposed method is compared with the HOG + HOG2, I3D, and MTUT methods. It can be seen that, for more complex datasets, the proposed method has a higher accuracy rate compared with the traditional HOG + HOG2 method, but the recognition rate is not obvious compared with the 3D and MTUT methods and even 1.23% lower than the MTUT accuracy rate, probably because, in complex datasets and data with large time spans, lighter models do not recognize connections between video clips very well.

In the RGB + optical flow information, the proposed method is compared with the 2S-CNNs, iDT, I3D, and MTUT methods. Although iDT is generally considered to be the best manual identification method at present, it can be seen that the method identification accuracy rate in this paper is 19.88% higher than that of the iDT method. In this mode, since the optical flow information contains a lot of gesture change information of the front and back frames at the same time, the method of this paper is higher than other methods in terms of accuracy.

**Figure 6:**
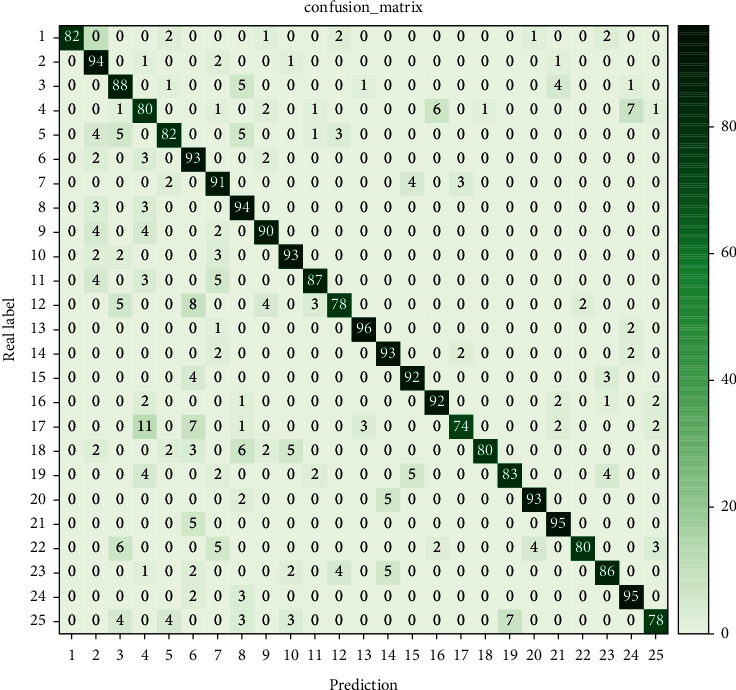
The recognition confusion matrix of the NVGesture dataset.

In the RGB + depth + optical flow information, the proposed method is compared with the R3DCNN, I3D, and MTUT methods. Among them, R3DCNN is the original method of this dataset, and it can be seen that the correct rate of the proposed method is 2.57% higher than that of the original method and 0.69% higher than that of the I3D method. Although the accuracy of the proposed method is 0.56% lower than that of the latest MTUT method, the results are within the acceptable range because the model is relatively simple in feature fusion. On this basis, the false positive rate of each gesture in the NVGesture dataset is relatively average, the average false error rate is 0.51%, and the recognition confusion matrix is shown in Figure 6.

In summary, the recognition accuracy rate of the proposed method on the NVGesture dataset has achieved a high level.

## 5. Conclusion

In order to further improve the performance of gesture recognition without relying on manual feature extraction, this paper proposes a gesture recognition method based on a deep learning model based on space-time feature fusion. In this model, features of gesture data are automatically extracted by convolutional neural networks. Then, long short-term memory (LSTM) networks were used to learn the correlation advantages of time series data. The SoftMax classifier is employed to classify multimodal gestures. Experimental results on the two dynamic gesture datasets of VIVA and NVGesture demonstrate that, compared with other comparison methods, the proposed model converges faster and the gesture recognition performance is better. In order to achieve the practical application of this algorithm, the subsequent work will analyze and improve the efficiency of the algorithm.

## Figures and Tables

**Table 1:** Experimental parameter settings.

Parameter	Options
Initialize the weight coefficient of CNN layer	Kaiming method
LSTM layer weight coefficient initialization	Orthogonal method
The weight coefficient of the full connection layer is initialized	Kaiming method
Optimizer	Adam optimizer
Loss function	Cross entropy
Initial learning rate	0.001
Sample sequence size	24 × 410
Number of training set samples	20088
Number of samples in test set	2232
Number of training wheels	20
Batch size	500
Leaky ReLU divisor	0.1

**Table 2:** The accuracy results on the VIVA dataset.

Method	Accuracy
HOG + HOG2 [35]	65.51
CNN : LRN [15]	75.41
CNN : LRN : HRN [15]	78.51
C3D [14]	78.41
I3D [32]	84.11
MTUT [36]	87.09
3D-Dense (a)	89.22
Res3D + TCNs (b)	86.98
T3D-Dense + TCNs (c)	91.74
**Proposed**	**92.55**

**Table 3:** The accuracy results on the NVGesture dataset.

Methods	Fusion model	Accuracy
HOG + HOG2 [35]	RGB + depth	37.91
I3D [32]	RGB + depth	84.83
MTUT [36]	RGB + depth	87.11
Proposed	RGB + depth	85.88
2S-CNNs	RGB + Opt.flow	66.61
iDT [37]	RGB + Opt.flow	74.41
I3D [32]	RGB + Opt.flow	85.44
MTUT [36]	RGB + Opt.flow	86.49
Proposed	RGB + Opt.flow	87.22
R3DCNN [6]	RGB + depth + Opt.flow	84.81
I3D [32]	RGB + depth + Opt.flow	86.69
**Proposed**	**RGB** **+** **depth** **+** **Opt.flow**	**87.38**

## Data Availability

The labeled datasets used to support the findings of this study are available from the corresponding author upon request.
